# PaO_2_/FiO_2_ and IL-6 are risk factors of mortality for intensive care COVID-19 patients

**DOI:** 10.1038/s41598-021-86676-3

**Published:** 2021-04-01

**Authors:** Yanli Gu, Donghui Wang, Cen Chen, Wanjun Lu, Hongbing Liu, Tangfeng Lv, Yong Song, Fang Zhang

**Affiliations:** 1 Department of Respiratory and Critical Care Medicine, Jinling Hospital, Nanjing Medical University, Nanjing, 210002 China; 2 Department of Respiratory and Critical Care Medicine, The First School of Clinical Medicine, Jinling Hospital, Southern Medical University, Nanjing, 210002 China; 3 Department of Respiratory and Critical Care Medicine, Jinling Hospital, Medical School of Nanjing University, Nanjing, 210002 China

**Keywords:** Medical research, Risk factors

## Abstract

To identify the risk factors of mortality for the coronavirus disease 19 (COVID-19) patients admitted to intensive care units (ICUs) through a retrospective analysis. The demographic, clinical, laboratory, and chest imaging data of patients admitted to the ICU of Huoshenshan Hospital from February 10 to April 10, 2020 were retrospectively analyzed. Student's t-test and Chi-square test were used to compare the continuous and categorical variables, respectively. The logistic regression model was employed to ascertain the risk factors of mortality. This retrospective study involved 123 patients, including 64 dead and 59 survivors. Among them, 57 people were tested for interleukin-6 (IL-6) (20 died and 37 survived). In all included patients, the oxygenation index (PaO_2_/FiO_2_) was identified as an independent risk factor (odd ratio [OR] = 0.96, 95% confidence interval [CI]: 0.928–0.994, *p* = 0.021). The area under the curve (AUC) was 0.895 (95% CI: 0.826–0.943, *p* < 0.0001). Among the patients tested for IL-6, the PaO_2_/FiO_2_ (OR = 0.955, 95%CI: 0.915–0.996, *p* = 0.032) and IL-6 (OR = 1.013, 95%CI: 1.001–1.025, *p* = 0.028) were identified as independent risk factors. The AUC was 0.9 (95% CI: 0.791–0.964, *p* < 0.0001) for IL-6 and 0.865 (95% CI: 0.748–0.941, *p* < 0.0001) for PaO_2_/FiO_2_. PaO_2_/FiO_2_ and IL-6 could potentially serve as independent risk factors for predicting death in COVID-19 patients requiring intensive care.

## Introduction

Since the first case was diagnosed in Wuhan, China, the coronavirus disease 2019 (COVID-19) caused by severe acute respiratory syndrome-coronavirus-2 (SARS-CoV-2) has spread across the world. SARS‐CoV‐2, is the seventh human coronavirus and belongs to β coronavirus. Six coronaviruses have infected humans previously, including SARS‐CoV‐1 and Middle East respiratory syndrome (MERS)‐CoV in 2003 and 2012, respectively. However, the pandemic caused by SARS‐CoV‐2 is unprecedented. The mortality rates of SARS and MERS were > 10% and 35%, respectively^1,2^, while it is only 3.12–5.43% among the hospitalized COVID-19 patients according to the data from National Health Commission of the People's Republic of China and World Health Organization.

Although the mortality rate of COVID-19 is lower than that of SARS and MERS, the overall number of deaths is higher owing to the greater number of infections. As of September 14, 2020, China reported 85,202 cases of infection and 4634 deaths^3^. Meanwhile, a whopping 28,918,900 cases of infection and 922,252 deaths have been reported globally from this disease^4^. The clinical data from Wuhan, China, indicate that 17.7–32% of the patients required ICU admission, and the mortality rate of the critically ill patients was as high as 49–61.5%^5,6^. Early identification of individuals at a high risk of mortality may help reduce the mortality rate associated with this disease.

The clinical manifestations of COVID-19 include asymptomatic infections, mild upper respiratory symptoms, and respiratory failure requiring advanced life support^7,8^. The severity of the disease has been classified into mild, common, severe, and critical according to the guidelines for the diagnosis and treatment of COVID-19 pneumonia published by the National Health Commission of China^9^. A significant proportion of the severe and critically ill patients require intensive care and have high mortality rates. If patients with a high risk of mortality can be identified early upon ICU admission, it would be helpful in focusing the treatment efforts on these patients toward reducing the mortality rate from COVID-19.

In this study, we retrospectively analyzed the clinical data of 123 patients admitted to the ICU of Huoshenshan Hospital, a specialized COVID-19 hospital in Wuhan, China, to identify the risk factors of mortality.

## Results

### Demographic, clinical, laboratory and chest imaging data of all ICU patients

As shown in Table 1, 64 cases (44 men, 20 women) in the deceased group and 59 cases (34 men, 25 women) in the surviving group were included from February 10 to April 10, 2020. The mean time from illness onset to ICU admission was 22.38 days. No statistical difference was observed in age, sex, and underlying diseases between the deceased and surviving groups. The most common symptom of the patients was fever, followed by cough, dyspnea, fatigue, chest tightness, muscle soreness, and poor appetite. Except fatigue (*p* = 0.011), these symptoms were similar between the two groups. The deceased patients had a higher heart rate (*p* = 0.003) and respiratory rate (*p* = 0.009), while no statistically significant difference was observed in the body temperature, systolic blood pressure (SBP), and diastolic blood pressure (DBP) (*p* > 0.05 in all instances) at ICU admission. As for the laboratory findings, there was a significant difference in the counts of leukocytes (*p* = 0.001), neutrophils (*p* = 0.001), lymphocytes (*p* = 0.029), platelets (*p* = 0.008), neutrophil–lymphocyte ratio (NLR) (*p* = 0.001), albumin (*p* = 0.001), urea nitrogen (*p* = 0.005), serum chloride (*p* = 0.029), myoglobin (*p* = 0.004), brain natriuretic peptide (BNP) (*p* = 0.021), D-dimer (*p* = 0.023), lactate dehydrogenase (LDH) (*p* < 0.0001), C reactive protein (CRP) (*p* < 0.0001), and procalcitonin (PCT) (*p* = 0.025) levels between the deceased and surviving group. When compared with the surviving patients, the oxygenation index (PaO_2_/FiO_2_) of the deceased patients was much lower (*p* < 0.0001), whereas the PaCO_2_ level was not significantly different (*p* = 0.821). In addition, chest imaging severity was also significantly different between the two groups. Since only 57 patients were tested for IL-6, IL-6 was not included in the analysis of 123 patients.

**Table 1:** Clinical characteristics of intensive care COVID-19 patients.

Characteristic	Total (n = 123)	Deceased group (n = 64)	Surviving group (n = 59)	*p* value
Age, year	70.2 ± 11.58	70.97 ± 11.17	69.37 ± 12.05	0.447
**Sex, n (%)**				0.261
Male	78(63.4)	44(68.7)	34(57.6)	
Female	45(36.6)	20(31.3)	25(42.4)	
**Underlying disease, n (%)**				
Hypertension	67(54.5)	32(50)	35(59.3)	0.366
Diabetes mellitus	28(22.8)	19(30)	9(15.3)	0.084
Coronary heart disease	14(11.4)	11(17.2)	3(5)	0.064
Cerebrovascular disease	12(10)	7(11)	5(8.5)	0.765
COPD	13(10.6)	7(11)	6(10.2)	1
Hepatic/renal insufficiency	14(11.4)	7(11)	7(11.9)	1
**Symptoms, n (%)**				
Fever	91(74)	48(75)	43(72.9)	0.839
Cough	90(73.2)	44(68.8)	46(78)	0.31
Dyspnea	75(61)	43(67.2)	32(54.2)	0.195
Chest tightness	32(26)	14(21.9)	18(30.5)	0.309
Fatigue	69(56.1)	43(67.2)	26(44.1)	0.011
Poor appetite	17(13.8)	7(10.9)	10(16.9)	0.435
Muscle soreness	29(23.6)	18(28.1)	11(18.6)	0.288
**Vital signs**				
Temperature, ℃	36.61 ± 1.06	36.64 ± 1.4	36.57 ± 0.49	0.72
Heart rate, beats per minute	95.38 ± 17.2	99.81 ± 18.19	90.58 ± 14.77	0.003
Respiratory rate, beats per minute	25.46 ± 6.91	27 ± 7.43	23.78 ± 5.92	0.009
Systolic blood pressure, mmHg	136.2 ± 22.11	134.63 ± 25.29	137.9 ± 18.11	0.414
Diastolic blood pressure, mmHg	79.59 ± 15.44	78.38 ± 14.73	80.92 ± 16.2	0.364
**Laboratory findings**				
Leucocyte count, *10^9/L	11.28 ± 8.13	13.56 ± 10.02	8.8 ± 4.24	0.001
Neutrophil count, *10^9/L	9.71 ± 7.89	11.95 ± 9.62	7.28 ± 4.34	0.001
Lymphocyte count, *10^9/L	0.82 ± 0.58	0.71 ± 0.43	0.94 ± 0.7	0.029
NLR, %	16.76 ± 14.42	20.96 ± 14.74	12.28 ± 12.72	0.001
Eosinophil count, *10^9/L	0.09 ± 0.16	0.07 ± 0.16	0.11 ± 0.16	0.132
Hemoglobin, g/L	114.68 ± 21.81	116.14 ± 23.6	113.07 ± 19.72	0.439
Platelet, *10^9/L	190.77 ± 108.33	166.17 ± 117.43	217.46 ± 91.17	0.008
Albumin, g/L	31.88 ± 6.21	30.06 ± 5.04	33.89 ± 6.79	0.001
Globulin, g/L	28.17 ± 5	27.97 ± 4.64	28.39 ± 5.4	0.644
ALT, IU/L	48.6 ± 83.77	60.1 ± 107.97	35.89 ± 41.12	0.114
Creatinine, umol/L	108.14 ± 155.46	112.76 ± 103.22	103.21 ± 197.44	0.736
Urea nitrogen, mmol/L	9.65 ± 7.33	11.44 ± 7.5	7.74 ± 6.69	0.005
Serum sodium, mmol/L	142.42 ± 10.57	143.6 ± 13.74	141.08 ± 4.84	0.181
Serum potassium, mmol/L	4.27 ± 0.68	4.35 ± 0.7	4.19 ± 0.66	0.208
Serum chloride, mmol/L	104.77 ± 6.24	105.94 ± 6.86	103.47 ± 5.22	0.029
Serum calcium, mmol/L	2.02 ± 0.18	1.99 ± 0.2	2.06 ± 0.15	0.051
Myoglobin, ng/ml	233.14 ± 659.61	421.04 ± 878.74	32.14 ± 65.04	0.004
Hypersensitive troponin I, ng/ml	0.36 ± 1.28	0.6 ± 1.72	0.12 ± 0.49	0.086
BNP, pg/ml	290.01 ± 599.91	411.57 ± 749.64	146.83 ± 300.83	0.021
APTT, s	30.72 ± 8.14	32.09 ± 9.84	29.28 ± 5.6	0.063
Thrombin time, s	17.68 ± 6.25	18.79 ± 8.34	16.51 ± 2.2	0.052
D-dimer, mg/L	5.35 ± 6.39	6.69 ± 6.97	3.91 ± 5.4	0.023
LDH, IU/L	383.96 ± 189.85	476.04 ± 205.33	291.89 ± 115.4	< 0.0001
CRP, mg/L	80.45 ± 77.84	111.77 ± 81.99	46.39 ± 56.39	< 0.0001
PCT, ng/ml	0.76 ± 2.44	1.31 ± 3.34	0.19 ± 0.19	0.025
PaO2/FiO2, mmHg	172.98 ± 119.38	108.06 ± 50.29	243.39 ± 132.43	< 0.0001
PaCO2, mmHg	40.16 ± 10.51	40.34 ± 12.82	39.92 ± 6.55	0.821
**Chest X-ray severity, n (%)**				< 0.0001
Mild	41(33.33)	16(25)	25(42.37)	
Moderate	38(30.89)	14(21.88)	24(40.68)	
Severe	41(33.33)	32(50)	9(15.25)	
Time from illness onset to ICU admission, days	22.38 ± 3.54	20.11 ± 11.39	24.8 ± 15.26	0.059

Data are presented as mean ± SD or number (%).

COPD, chronic obstructive pulmonary disease; ICU, intensive care unit; NLR, neutrophil–lymphocyte ratio; ALT, alanine aminotransferase; AST, aspartate transaminase; A/G ratio, white/globule ratio; BNP, brain natriuretic peptide; APTT, activated partial thromboplastin time; LDH, lactate dehydrogenase; IL-6, interleukin-6; CRP, C-reactive protein; PCT, procalcitonin; PaO2, arterial partial pressure of oxygen; FiO2, oxygen concentration; PaCO2, arterial partial pressure of carbon dioxide.

### Risk factors of mortality of all ICU patients

**Figure 1:**
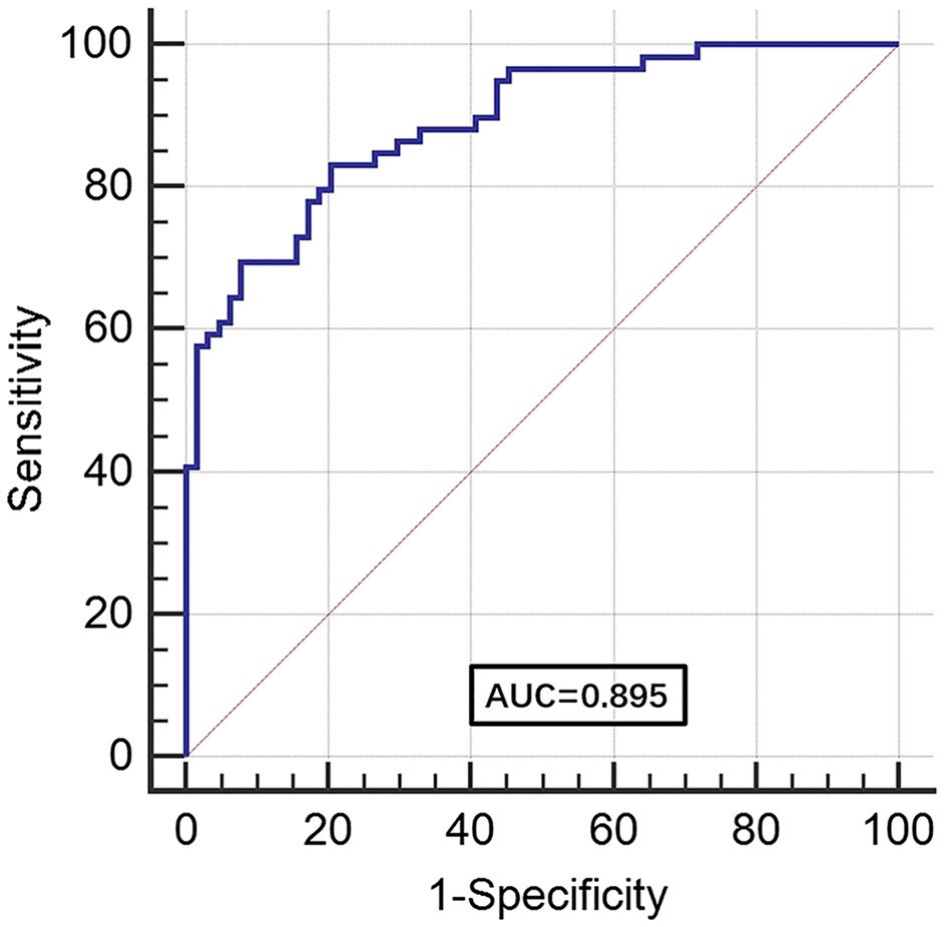
The AUC of PaO2/FiO2 in all patients (AUC = 0.895 (95% CI: 0.826–0.943, *p* < 0.0001)). Abbreviations: AUC: area under the curve; PaO2/FiO2: oxygenation index.

From the clinical data and laboratory findings of these patients, we selected indicators with *p* < 0.05, including heart rate, respiratory rate, leukocyte, neutrophil, lymphocyte, NLR, platelet, albumin, urea nitrogen, serum chloride, myoglobin, BNP, D-dimer, LDH, CRP, PCT, PaO_2_/FiO_2_, and chest imaging severity, and incorporated them into the logistic regression model. As shown in Table 2, univariate logistic regression analysis revealed that the above indicators were associated with death in COVID-19 patients, except for the respiratory rate. The abovementioned indicators were then further incorporated into multivariable analysis. PaO_2_/FiO_2_ (OR = 0.96, 95% CI: 0.928–0.994, *p* = 0.021) were found to be an independent risk factor of mortality for the COVID-19 patients. We thereby drew the receiver operator characteristic (ROC) curve of PaO_2_/FiO2. As shown in Fig. 1, the AUC was 0.895 (95% CI: 0.826–0.943, *p* < 0.0001), with a sensitivity of 81.2% and a specificity of 83.1% when the cut-off value was 152.86 mmHg.

**Table 2:** Risk factors associated with mortality for intensive care COVID-19 patients.

Characteristic	Univariable OR (95%CI)	*p* value	Multivariable OR (95%CI)	*p* value
Heart rate, beats per minute	1.035(1.011–1.059)	0.004		
**Respiratory rate, beats per minute**				
≤ 25	1(ref)			
> 25	2.047(0.969–4.325)	0.061		
Leucocyte count, *10^9/L				
≤ 10	1(ref)			
> 10	2.749(1.31–5.769)	0.007		
Neutrophil count, *10^9/L				
≤ 6.3	1(ref)			
> 6.3	2.427(1.168–5.042)	0.017		
Lymphocyte count, *10^9/L	2.391(1.053–5.43)	0.037		
NLR, %	1.054(1.02–1.089)	0.002		
Platelet, *10^9/L	1.005(1.001–1.008)	0.011		
Albumin, g/L	1.171(1.067–1.284)	0.001		
**Urea nitrogen, mmol/L**				
≤ 8.8	1(ref)			
> 8.8	4.592(2.054–10.266)	< 0.0001		
Serum chloride, mmol/L	1.07(1.004–1.14)	0.036		
Myoglobin, ng/ml	1.01(1.003–1.017)	0.005		
BNP, pg/ml	1.002(1–1.003)	0.028		
D-dimer, mg/L	1.091(1.006–1.182)	0.034		
**LDH, IU/L**				
≤ 250	1(ref)			
> 250	6.72(2.479–18.219)	< 0.0001		
**CRP, mg/L**				
≤ 10	1(ref)			
> 10	5.444(2.004–14.788)	0.001		
PCT, ng/ml	31.74(4.16–241.89)	0.001		
PaO2/FiO2, mmHg	0.974 (0.965–0.983)	< 0.0001	0.96(0.928–0.994)	0.021
**Chest imaging severity**				
Mild	1(ref)			
Moderate	5.556(2.106–14.653)	0.001		
Severe	6.095(2.263–16.414)	< 0.0001		

NLR, neutrophil–lymphocyte ratio; BNP, brain natriuretic peptide; LDH, lactate dehydrogenase; CRP, C-reactive protein; PCT, procalcitonin; PaO2, arterial partial pressure of oxygen; FiO2, oxygen concentration.

### Risk factors of mortality in the subgroup analysis

Several studies had reported that IL-6 was involved in the pathophysiological process of COVID-19; therefore, we performed a subgroup analysis in patients who were tested for IL-6 to explore whether IL-6 could be an independent risk factor at our center. A total of 57 patients tested for IL-6, among whom 20 cases (14 men, 6 women) in the deceased group and 37 cases (24 men, 13 women) in the surviving group were included. The clinical characteristics of the included patients have been shown in the Supplementary Material 1 (Tables S1). After multivariate logistic regression analysis, both IL-6 (OR = 1.013, 95%CI: 1.001–1.025, *p* = 0.028) and PaO_2_/FiO_2_ (OR = 0.955, 95%CI: 0.915–0.996, *p* = 0.032) were identified as independent risk factors (Supplementary Material 1, Table S2). Among patients not tested for IL-6, the PaO_2_/FiO_2_ (OR = 0.976, 95%CI: 0.953–0.998, *p* = 0.037) was still an independent risk factor (Supplementary Material 1, Tables S3 and S4).

**Figure 2:**
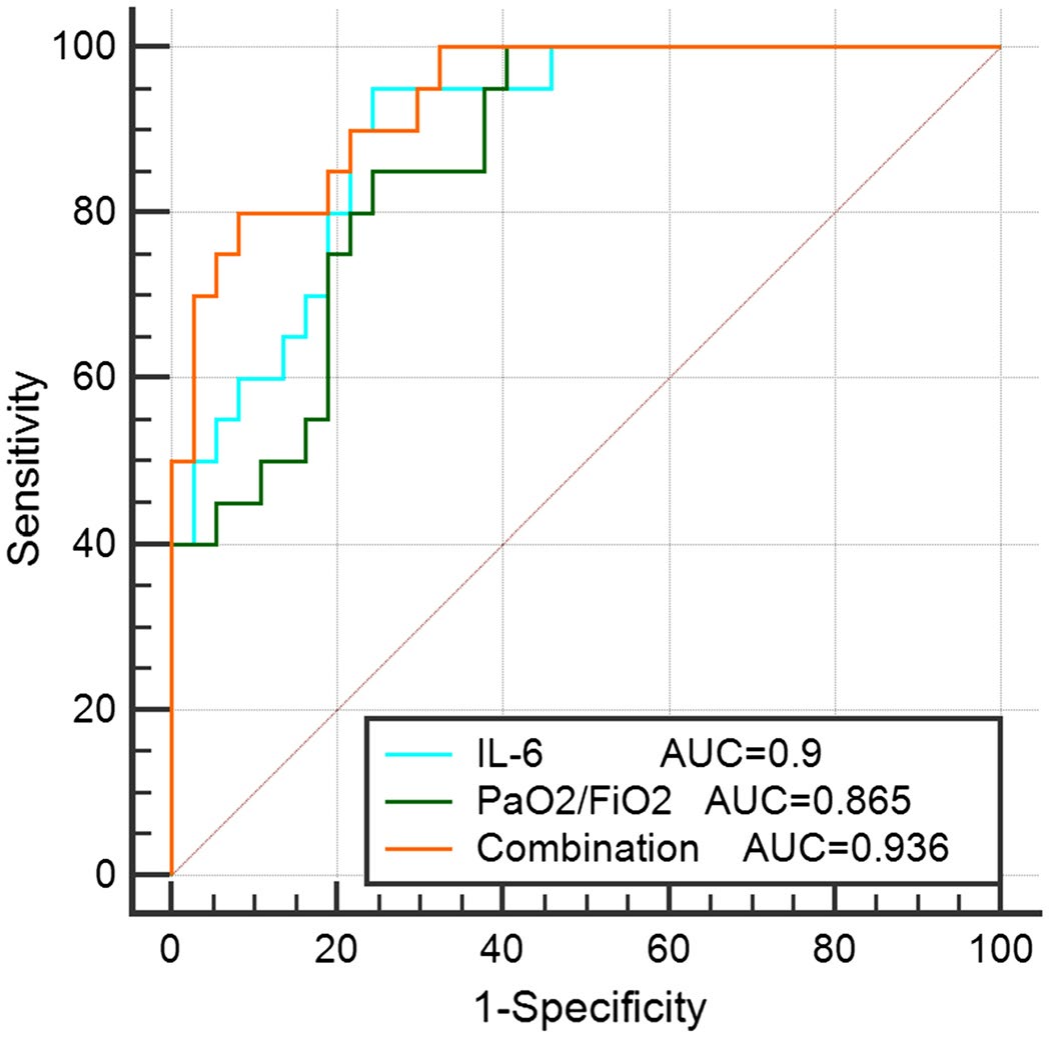
The AUC of IL-6 and PaO2/FiO2 and their combination in patients tested for IL-6. The AUC of IL-6 and PaO2/FiO2 were 0.9 (95% CI: 0.791–0.964, *p* < 0.0001) and 0.865 (95% CI: 0.748–0.941, *p* < 0.0001) respectively. The combination ROC curve of IL-6 and PaO2/FiO2 had an AUC of 0.936 (95%CI: 0.839–0.984), which were not statistically significant when compared with IL-6 (*p* = 0.319) or PaO2/FiO2 (*p* = 0.0502). Abbreviations: AUC: area under the curve; PaO2/FiO2: oxygenation index; IL-6: interleukin-6.

**Figure 3:**
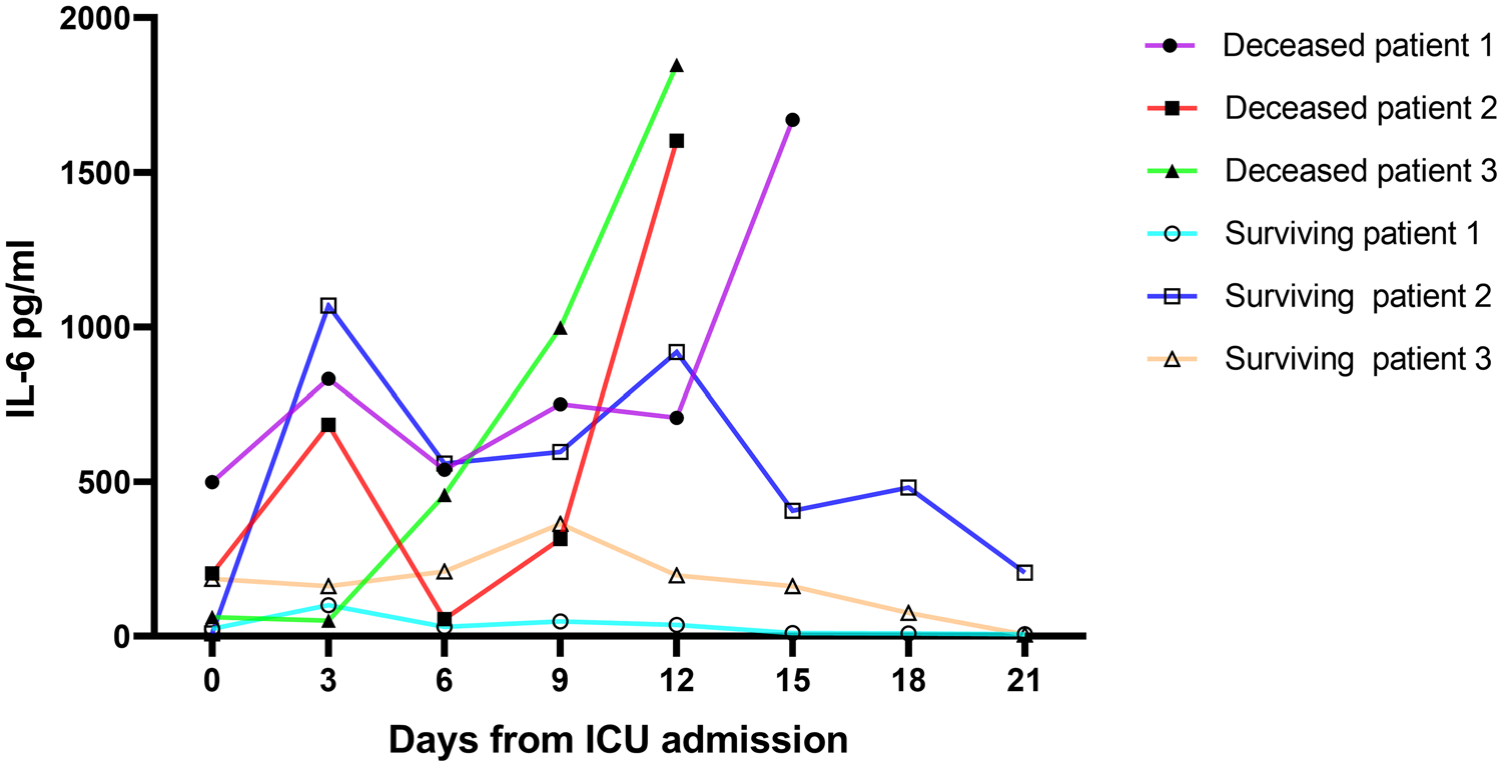
The variation trend of IL-6 levels in six patients (3 in deceased group and 3 in surviving group) during hospitalization. Abbreviations: IL-6: interleukin-6.

We further drew the receiver operator characteristic (ROC) curves of IL-6 and PaO2/FiO2 and their combination curve among patients tested for IL-6. As shown in Fig. 2, the AUCs of IL-6 and PaO2/FiO2 were 0.9 (95% CI: 0.791–0.964, *p* < 0.0001) and 0.865 (95% CI: 0.748–0.941, *p* < 0.0001), respectively. The cut-off value of IL-6 was 24.24 pg/mL, with a sensitivity of 95% and a specificity of 75.68%, while the cut-off value of PaO2/FiO2 was 167 mmHg, with a sensitivity of 85% and a specificity of 75.68%. The combination ROC curve of IL-6 and PaO2/FiO2 had an AUC of 0.936 (95%CI: 0.839–0.984), which were not statistically significant when compared with IL-6 (*p* = 0.319) or PaO2/FiO2 (*p* = 0.0502). The ROC curve of PaO2/FiO2 in patients not tested for IL-6 was shown in Supplementary Material 1 (Figure S1). Furthermore, we observed the variation trend of IL-6 levels in 6 patients (3 in the deceased group and 3 in the surviving group) whose plasma IL-6 was tested > 5-times during hospitalization. The levels of IL-6 decreased gradually as the condition of the patients improved in the surviving group, while it increased as the patients’ condition deteriorated in the deceased group (Fig. 3).

## Discussion

This retrospective study confirmed several factors related to the death of COVID-19 patients receiving intensive care, including heart rate, leukocyte count, neutrophil count, lymphocyte count, NLR, platelet count, albumin level, urea nitrogen level, serum chloride level, myoglobin level, BNP level, D-dimer level, LDH level, CRP level, PCT level, PaO_2_/FiO_2_ level, and chest imaging severity, among which the level of PaO_2_/FiO_2_ was an independent risk factor. In fact, IL-6 was also an independent risk factor when considered.

PaO_2_/FiO_2_, the most commonly used oxygenation index, is included in the sepsis management guideline^10^ and acute respiratory distress syndrome (ARDS)^11^ although it may overestimate the incidence of ARDS and underestimate ARDS mortality^12^. Lung is the most important organ invaded by SARS-CoV-2. Several COVID-19 patients are characterized by hypoxia and respiratory distress. Post-mortem histological examination revealed hyaline membranes, and mixed inflammatory cell infiltration of the interstitium, alveoli, and perivascular areas^13^, which are consistent with the characteristics of ARDS. In this study, PaO2/FiO2 was an independent predictor of COVID-19 death irrespective of considering IL-6.

Evidence have shown that cytokine release syndrome (CRS) plays an important role in the pathogenesis of COVID-19. IL-6, one of the inflammatory cytokines involved in CRS, was significantly elevated, which is the key driver of the inflammatory process in COVID-19^7,14^. Excessive IL-6 can lead to organ damage, such as increasing vascular permeability^15^ and decreasing myocardial contractility^16^. Consistent with a previous large retrospective cohort study^17^, we noted that IL-6 was another independent risk factor of mortality for COVID-19, with 95% sensitivity and 75.68% specificity at the cut-off value of 24.24 pg/mL. Despite the lack of statistical significance, the combined application of IL-6 and PaO_2_/FiO_2_ showed superior sensitivity and specificity. In addition, the level of IL-6 in the deceased group increased gradually, while it improved in the surviving group. This finding indicates that the disease prognosis could possibly be judged based on the changing trend of IL-6 value.

IL-6 activates downstream JAK signaling pathway by binding to either trans-membrane (*cis*-signaling) or soluble (*trans*-signaling) IL-6R^18^. Tocilizumab, a recombinant humanized monoclonal anti-IL‐6R antibody, can bind to both trans-membrane and soluble IL-6R to inhibit IL-6-mediated *cis-* and *trans-*signaling^19^. Studies had shown the efficacy of tocilizumab against COVID-19^20,21^. However, all of them are retrospective studies and the number of reported cases is small. Larger random control trials are needed in the future to confirm the therapeutic effect of tocilizumab on COVID-19.

This study has several limitations. First, the sample size was small in this single-center retrospective study; hence, the results should be validated with additional studies. Second, this is a retrospective study and not a prospective study. Pharmacological therapies implemented and the time of hospitalization may affect the laboratory values and PaO_2_/FiO_2_. We hence selected the laboratory findings on the day of ICU admission or the day after ICU admission to minimize the adverse impact of subsequent treatments. Third, because the imaging severity was not evaluated by computed tomography, the chest X-ray results may be inconsistent with the actual lung lesions. Finally, not all laboratory tests were performed in all patients, for instance, serum ferritin and T lymphocyte subpopulation. Therefore, their role in mortality could not be evaluated.

In conclusion, PaO2/FiO2 and IL-6 could potentially serve as independent risk factors for predicting death in COVID-19 patients requiring intensive care, and the prognosis of patients could possibly be judged according to the change in the trend of IL-6 level. Thus, clinicians might want to consider the aforementioned indicators and take active action to reduce the mortality of COVID-19.

## Methods

### Patients

From February 10 to April 10, 2020, a total of 123 patients were admitted to the ICU of Huoshenshan Hospital. Among these patients, 66 patients did not detect IL-6 while 57 patients did. SARS-CoV-2 infection was confirmed in all the patients by reverse transcription-polymerase chain reaction (RT-PCR). Patients admitted to the ICU had to satisfy any of the following criteria: 1. respiratory failure necessitating mechanical ventilation, 2. unstable vital signs requiring electrocardiographic monitoring, and 3. Presenting other complications such as gastrointestinal bleeding, heart failure, and renal failure also. This study was approved by the Ethics Committee of the Wuhan Huoshenshan Hospital, and informed consent was obtained from all individual participants or their families. The ethics committee approval number was HSSLL033. All methods were performed in accordance with the relevant guidelines and regulations.

### Data collection

All data were obtained from the electronic medical system and were independently checked by two researchers to ascertain their accuracy. Detailed demographic information, underlying diseases, clinical symptoms, vital signs, laboratory findings, and imaging severity of all the patients were recorded when they entered the ICU. Demographic information included age and sex. Underlying diseases included hypertension, diabetes mellitus, coronary heart disease, cerebrovascular disease (cerebral infarction/hemorrhage), COPD, and hepatic/renal insufficiency. Clinical symptoms included fever, cough, dyspnea, chest tightness, fatigue, poor appetite, and muscle soreness. Vital signs included body temperature, heart rate, respiratory rate, and blood pressure. Laboratory tests included blood routine, liver, kidney, heart, and coagulation indices; biological indicators were related to inflammation or infection, oxygenation index (PaO_2_/FiO_2_), and PaCO_2_. Some indicators were tested several times, but we selected the laboratory findings on the day of ICU admission or the day after ICU admission. According to the lung lesion range, the chest X-ray findings were divided into mild, moderate, and severe. Mild was defined as a lesion area involving 1–2 lung fields, moderate involving 3–4 lung fields, and severe involving 5–6 lung fields.

### Data analysis

The continuous and categorical variables were expressed as mean ± standard deviation (SD) and frequency, respectively. Student's t-test and Chi-square test were used to compare the continuous and categorical variables between the groups of deceased and surviving patients. To identify independent risk factors of mortality, univariate and multivariate logistic regression models were used. We included variables into univariate logistic analysis if their between-group differences were significant. Then variables that were significant in univariate logistic analysis were further incorporated into multivariate logistic analysis. Deceased group was defined as 0 and surviving group as 1 in logistic analysis. *p* < 0.05 (two-tailed) was considered to be statistically significant. All statistical analyses were performed using SPSS (version 25.0).

### Ethics approval

This study was approved by the Ethics Committee of the Wuhan Huoshenshan Hospital.

### Consent to participate

Informed consent was obtained from all individual participants or their families included in the study.

## Supplementary Information

Supplementary Information.
