# Supplementary material for: PaO2/FiO2 and IL-6 are risk factors of mortality for intensive care COVID-19 patients
Source: Sci Rep. 2021 Apr 1;11:7334. doi: 10.1038/s41598-021-86676-3 (PMC8016870; doi:10.1038/s41598-021-86676-3)
Supplement: Supplementary file 1 — Supplementary Information. [file 41598_2021_86676_MOESM1_ESM.pdf]

Supplementary Material 1

PaO₂/FiO₂ and IL-6 are risk factors of mortality for intensive care COVID-19 patients.

Authors: Yanli Gu[1], Donghui Wang[1], Cen Chen[2], Wanjun Lu[3], Hongbing Liu[3], Tangfeng Lv[3], Yong Song[1,3,*], Fang Zhang[1,3,*]

Table S1: Clinical characteristics of intensive care COVID-19 patients tested for IL-6.

Characteristic	Total (n=57)	Deceased group (n=20)	Surviving group (n=37)	P value
**Age, year**	70.89±11.79	71.7±9.921	70.46±12.8	0.708
**Sex, n（%）**				0.775
Male	38(66.67)	14(70)	24(64.86)	
Female	19(33.33)	6(30)	13(35.14)	
**Underlying disease, n（%）**				
Hypertension	31(54.39)	11(55)	20(54.05)	1
Diabetes mellitus	14(24.56)	8(40)	6(16.22)	0.059
Coronary heart disease	5(8.77)	3(15)	2(5.41)	0.332
Cerebrovascular disease	9(15.79)	5(25)	4(10.81)	0.253
COPD	6(10.53)	1(5)	5(13.51)	0.41
Hepatic/renal insufficiency	7(12.28)	3(15)	4(10.81)	0.687
**Symptoms, n（%）**				
Fever	34(59.65)	11(55)	23(62.16)	0.778
Cough	41(71.93)	13(65)	28(75.68)	0.538
Dyspnea	34(59.65)	12(60)	22(59.46)	1
Chest tightness	16(28.07)	5(25)	11(29.73)	0.767
Fatigue	24(42.11)	11(55)	13(43.24)	0.77
Poor appetite	11(19.3)	3(15)	8(21.62)	0.73
Muscle soreness	7(12.28)	4(20)	3(11.11)	0.438
**Vital Signs**				
Temperature ℃	36.55±0.42	36.66±0.52	36.49±0.35	0.154
Heart rate, beats per minute	91.12±16.36	96.5±17.08	88.22±15.41	0.068
Respiratory rate, beats per minute	24.6±6.26	26.05±6.13	23.81±6.27	0.2
Systolic blood pressure, mmHg	138.6±21.51	139.05±27.17	138.35±18.17	0.908
**Laboratory findings**				
Leucocyte count, *10^9/L	10.29±5.11	12.32±5.33	9.2±4.71	0.027
Neutrophil count, *10^9/L	8.89±5.17	11.14±5.1	7.67±4.85	0.014
Lymphocyte count, *10^9/L	0.81±0.39	0.63±0.25	0.91±0.42	0.003
NLR	14.57±12.41	11.14±5.1	7.67±4.85	0.049
Eosinophil count, *10^9/L	0.1±0.16	0.02±0.03	0.14±0.19	0.001
Hemoglobin, g/L	110.89±18.28	109.25±19.85	111.78±17.59	0.622

Platelet, *10^9/L	206.09±121.78	191.7±156.32	213.86±99.97	0.517
Albumin, g/L	33.01±3.99	31.71±3.96	33.71±3.88	0.07
Globulin, g/L	27.88±4.84	27.78±4.95	27.93±4.85	0.914
ALT, IU/L	42.71±82.42	70.78±134.63	27.54±18.11	0.169
Creatinine，umol/L	86.11±86.17	118.75±133.36	68.46±35.38	0.113
Urea nitrogen, mmol/L	8.6±6.85	11.66±9.54	6.95±4.11	0.047
Serum sodium, mmol/L	4.28±0.74	4.434±0.85	4.1849±0.67	0.235
Serum potassium, mmol/L	142.47±14.47	145.37±22.96	140.81±5.5	0.392
Serum chloride, mmol/L	102.52±6.85	102.85±8.35	102.34±5.94	0.792
Serum calcium, mmol/L	2.07±0.16	2.02±0.16	2.1±0.15	0.05
Myoglobin, ng/ml	189.06±745.03	475.27±1206.73	28.06±67.01	0.135
Hypersensitive troponin I, ng/ml	0.15±0.73	0.39±1.26	0.04±0.08	0.283
BNP, pg/ml	312.61±758.87	541.28±1136.33	167.79±309.48	0.177
APTT, s	30.96±10.13	35.22±14.59	28.7±5.81	0.075
Thrombin time, s	16.85±8.24	19.67±13.68	15.37±1.38	0.188
D-dimer, mg/L	5.03±5.89	5.8±5.16	4.63±6.23	0.485
LDH, IU/L	336.05±152.47	426.65±174.07	288.24±116.1	0.001
IL-6, pg/ml	339.47±980.67	884.36±1529.86	44.94±81.59	0.024
CRP, mg/L	65.99±67.73	103.65±69.71	45.07±57.53	0.001
PCT, ng/ml	0.54±1.61	1.19±2.71	0.21±0.19	0.169
PaO2/FiO2, mmHg	212.2±145.46	117.41±46.22	263.9±155.07	<0.0001
PaCO2, mmHg	42.3±13.82	44.47±19.55	40.77±7.75	0.442
**Chest X-ray severity, %**				0.01
Mild	20(37.04)	3(16.67)	17(47.22)	
Moderate	20(37.04)	6(33.33)	14(38.89)	
Severe	14(25.92)	9(50)	5(13.89)	
**Time from illness onset to ICU admission**	29.07±14.88	26.95±13.15	30.22±15.88	0.434

Data are presented as mean ± SD or number (%)

Abbreviation: COPD=chronic obstructive pulmonary disease; NLR=neutrophil-to-lymphocyte ratio. ALT=alanine aminotransferase. AST=aspartate transaminase. A/G ratio=white/globule ratio. BNP=brain natriuretic peptide. APTT=activated partial thromboplastin time. LDH=lactate dehydrogenase. IL-6=interleukin-6. CRP=C-reactive protein. PCT=procalcitonin. PaO2=arterial partial pressure of oxygen. FiO2=oxygen concentration. PaCO2=arterial partial pressure of carbon dioxide.

Table S2: Risk factors associated with mortality for intensive care COVID-19 patients tested for IL-6.

Characteristic	Univariable OR (95%CI)	P value	Multivariable OR (95%CI)	P value
Leucocyte count, *10^9/L				
≤10	1(ref)			
>10	2.769(0.903-8.493)	0.075		
Neutrophil count, *10^9/L				
≤6.3	1(ref)			
>6.3	3.4(0.953-12.134)	0.059		
Lymphocyte count, *10^9/L	0.093(0.014-0.641)	0.016		
Eosinophil count, *10^9/L				
≥0.02	1(ref)			
<0.02	3.125(1.011-9.662)	0.048		
Urea nitrogen, mmol/L				
≤8.8	1(ref)			
>8.8	2.966(0.913-9.637)	0.071		
LDH, IU/L				
≤250	1(ref)			
>250	4.772(1.181-19.273)	0.028		
IL-6, pg/ml	1.009(1.003-1.015)	0.003	1.013(1.001-1.025)	0.028
CRP, mg/L				
≤10	1(ref)			
>10	10.739(1.285-89.717)	0.028		
NLR	1.095(1.026-1.168)	0.006		
PaO2/FiO2, mmHg	0.977(0.964-0.99)	0.001	0.955(0.915-0.996)	0.032
Chest X-ray severity				
Mild	1(ref)			
Moderate	2.429(0.512-11.511)	0.264		
Severe	10.2(1.971-52.775)	0.006		

Abbreviations: LDH=lactate dehydrogenase. IL-6=interleukin-6. CRP=C-reactive protein. NLR=neutrophil-lymphocyte ratio. PaO2=arterial partial pressure of oxygen. FiO2=oxygen concentration.

Table S3: Clinical characteristics of intensive care COVID-19 patients not tested for IL-6.

Characteristic	Total (n=66)	Deceased group (n=44)	Surviving group (n=22)	P value
**Age, year**	69.61±11.45	70.64±11.79	67.55±10.7	0.305
**Sex, n（%）**				0.109
Male	40(60.61)	30(68.18)	10(45.45)	
Female	26(39.39)	14(31.82)	12(54.55)	
**Underlying disease, n（%）**				
Hypertension	36(54.55)	21(47.73)	15(68.18)	0.189
Diabetes mellitus	14(21.21)	11(25)	3(13.64)	0.354
Coronary heart disease	9(13.64)	8(18.18)	1(4.55)	0.253
Cerebrovascular disease	3(3.03)	2(4.55)	1(4.55)	1
COPD	7(10.61)	6(13.64)	1(4.55)	0.409
Hepatic/renal insufficiency	7(10.61)	4(9.09)	3(13.64)	0.678
**Symptoms, n（%）**				
Fever	57(86.36)	37(84.09)	20(90.91)	0.706
Cough	49(74.24)	31(70.45)	18(81.82)	0.549
Dyspnea	41(62.12)	31(70.45)	10(45.45)	0.062
Chest tightness	16(24.24)	9(20.45)	7(31.82)	0.367
Fatigue	45(68.18)	32(72.73)	13(59.09)	0.278
Poor appetite	6(9.09)	4(9.09)	2(9.09)	1
Muscle soreness	22(33.33)	14(31.82)	8(36.36)	0.785
**Vital Signs**				
Temperature, °C	36.66±1.4	36.64±1.66	36.71±0.65	0.834
Heart rate, beats per minute	99.06±17.19	101.32±18.66	94.55±13	0.132
Respiratory rate, beats per minute	26.20±7.39	27.43±7.98	23.73±5.42	0.054
Systolic blood pressure, mmHg	134.12±22.57	132.61±24.44	137.14±18.42	0.447
Diastolic blood pressure, mmHg	79.98±14.3	80.32±14.22	79.32±14.36	0.791
**Laboratory findings**				
Leucocyte count, *10^9/L	12.13±9.99	14.13±11.55	8.13±3.29	0.002
Neutrophil count, *10^9/L	10.41±9.62	12.31±11.12	6.61±3.31	0.003
Lymphocyte count, *10^9/L	0.82±0.71	0.74±0.49	0.98±1.01	0.2
NLR, %	18.69±15.82	21.9±16.86	12.42±11.5	0.021
Eosinophil count, *10^9/L	0.08±0.16	0.09±1.19	0.06±0.08	0.503
Hemoglobin, g/L	118±24.15	119.27±24.7	115.33±23.3	0.543
Platelet, *10^9/L	177.55±94.16	154.57±94.7	223.50±75.9	0.004
Albumin, g/L	30.87±7.59	29.3±5.35	34.24±10.35	0.015
Globulin, g/L	28.44±5.17	28.05±4.55	29.26±6.33	0.397
ALT, IU/L	53.93±85.27	55.13±94.51	51.36±63.03	0.871
Creatinine, umol/L	127.47±195.91	109.98±87.5	101.15±142.23	0.76
Urea nitrogen, mmol/L	10.57±7.66	11.34±6.47	0.98±9.57	0.264
Serum sodium, mmol/L	4.26±0.62	4.31±0.62	4.16±0.61	0.386

Serum potassium, mmol/L	142.37±5.22	142.75±5.87	141.56±3.49	0.404
Serum chloride, mmol/L	106.77±4.89	107.4±5.54	105.44±2.78	0.068
Serum calcium, mmol/L	1.97±0.19	1.97±0.22	1.98±0.12	0.99
Myoglobin, ng/ml	289.66±534.77	386.18±605.71	43.98±60.34	0.006
Hypersensitive troponin I, ng/ml	0.63±1.75	0.72±1.96	0.38±1.00	0.61
BNP, pg/ml	267.41±387.75	339.09±407.55	104.92±288.48	0.05
APTT, s	30.5±5.74	30.57±6.08	30.37±5.14	0.905
Thrombin time, s	18.47±3.33	18.36±3.88	18.68±1.78	0.74
D-dimer, mg/L	5.67±6.94	7.15±7.76	2.31±2.17	0.001
LDH, IU/L	430.19±211.18	501.4±217.52	298.47±116.84	<0.0001
CRP, mg/L	93.31±84.3	115.63±87.76	48.65±55.7	0.002
PCT, ng/ml	0.99±3.09	1.36±3.65	0.13±0.18	0.214
PaO2/FiO2, mmHg	138.84±77.25	103.81±51.98	208.9±72.28	<0.0001
PaCO2, mmHg	38.66±7.13	38.56±8.14	38.87±4.65	0.843
**Chest X-ray severity, n (%)**				0.015
Mild	21(31.82)	13(29.55)	8(36.36)	
Moderate	18(27.27)	8(18.18)	10(45.45)	
Severe	27(40.91)	23(52.27)	4(18.18)	
**Time from illness onset to ICU admission, days**	16.51±8.86	16.93±8.98	15.68±8.77	0.595

Data are presented as mean ± SD or number (%)

Abbreviation: COPD=chronic obstructive pulmonary disease. ICU=intensive care unit. NLR=neutrophil-lymphocyte ratio. ALT=alanine aminotransferase. AST=aspartate transaminase. A/G ratio=white/globule ratio. BNP=brain natriuretic peptide. APTT=activated partial thromboplastin time. LDH=lactate dehydrogenase. IL-6=interleukin-6. CRP=C-reactive protein. PCT=procalcitonin. PaO2=arterial partial pressure of oxygen. FiO2=oxygen concentration. PaCO2=arterial partial pressure of carbon dioxide.

Table S4: Risk factors associated with mortality for intensive care COVID-19 patients not tested for IL-6.

Characteristic	Univariable OR (95%CI)	P value	Multivariable OR (95%CI)	P value
Leucocyte count, *10^9/L				
≤10	1(ref)			
>10	2.921(0.963-8.855)	0.058		
Neutrophil count, *10^9/L				
≤6.3	1(ref)			
>6.3	3.403(1.152-10.053)	0.027		
NLR	1.053(1.006-1.103)	0.026		
Platelet, *10^9/L	0.991(0.985-0.998)	0.007		
Myoglobin, ng/ml	1.007(0.998-1.017)	0.139		
Albumin, g/L	0.89(0.793-0.998)	0.046		
LDH, IU/L				
≤250	1(ref)			
>250	7.556(1.718-33.229)	0.007		
CRP, mg/L				
≤10	1(ref)			
>10	4.554(1.262-16.439)	0.021		
D-dimer, mg/L	1.365(1.063-1.754)	0.015		
PaO2/FiO2, mmHg	0.972(0.959-0.985)	<0.0001	0.976(0.953-0.998)	0.037
Chest X-ray severity				
Mild	1(ref)			
Moderate	3.538(0.891-14.058)	0.073		
Severe	7.187(1.753-29.477)	0.006		

Abbreviations: NLR=neutrophil-lymphocyte ratio. LDH=lactate dehydrogenase. CRP=C-reactive protein. PaO2=arterial partial pressure of oxygen. FiO2=oxygen concentration.

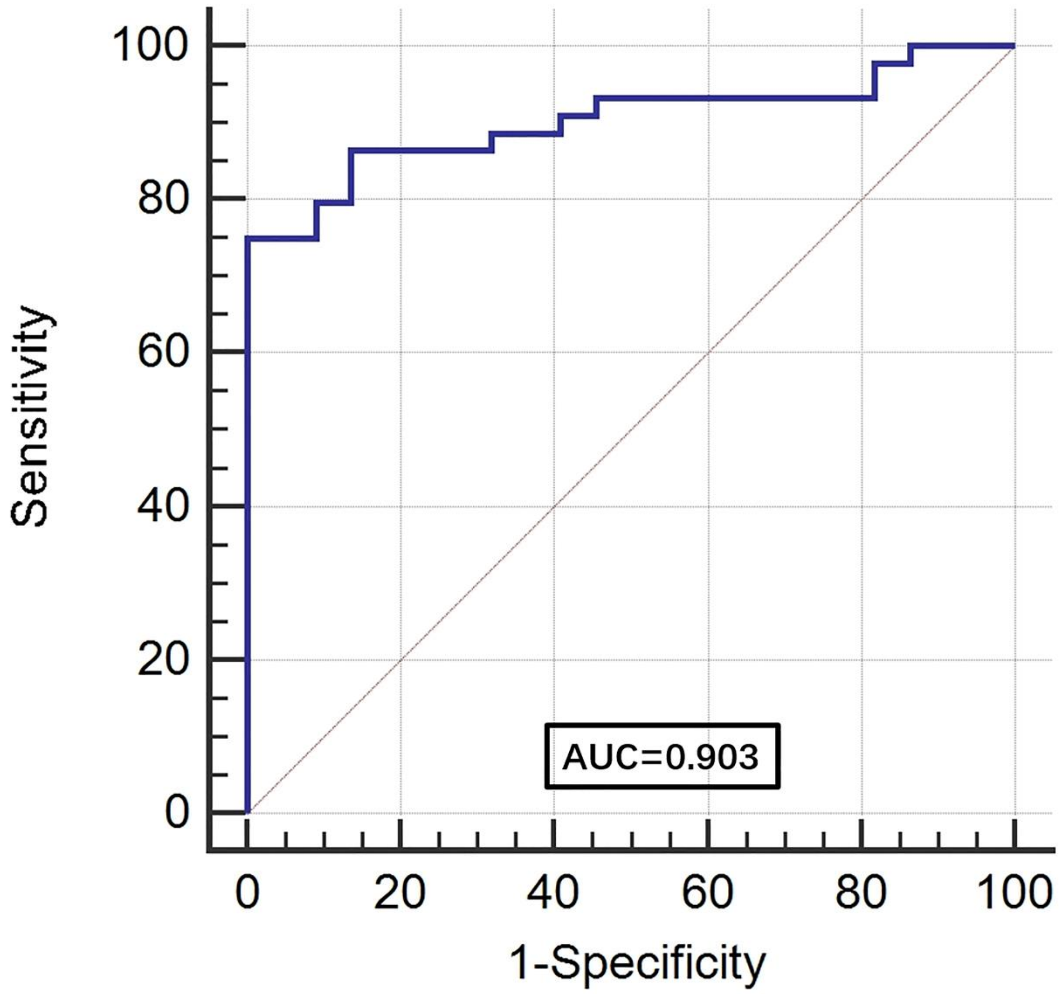

Figure S1: The AUC of PaO2/FiO2 in patients not tested for IL-6. (AUC=0.903 (95% CI: 0.805 - 0.962, p < 0.0001)). Abbreviations: AUC: area under the curve; PaO2/FiO2: oxygenation index; IL-6: interleukin-6.
